# SETD2 regulates gene transcription patterns and is associated with radiosensitivity in lung adenocarcinoma

**DOI:** 10.3389/fgene.2022.935601

**Published:** 2022-08-10

**Authors:** Zihang Zeng, Jianguo Zhang, Jiali Li, Yangyi Li, Zhengrong Huang, Linzhi Han, Conghua Xie, Yan Gong

**Affiliations:** ^1^ Department of Radiation and Medical Oncology, Zhongnan Hospital of Wuhan University, Wuhan, China; ^2^ Department of Biological Repositories, Zhongnan Hospital of Wuhan University, Wuhan, China; ^3^ Hubei Key Laboratory of Tumor Biological Behaviors, Zhongnan Hospital of Wuhan University, Wuhan, China; ^4^ Hubei Cancer Clinical Study Center, Zhongnan Hospital of Wuhan University, Wuhan, China; ^5^ Tumor Precision Diagnosis and Treatment Technology and Translational Medicine, Hubei Engineering Research Center, Zhongnan Hospital of Wuhan University, Wuhan, China

**Keywords:** lung adenocarcinoma, radiosensitivity, SETD2, DNA damage response, multi-omics, prognosis, epigenetic

## Abstract

Lung adenocarcinoma (LUAD) has high morbidity and mortality worldwide, and its prognosis remains unsatisfactory. Identification of epigenetic biomarkers associated with radiosensitivity is beneficial for precision medicine in LUAD patients. SETD2 is important in repairing DNA double-strand breaks and maintaining chromatin integrity. Our studies established a comprehensive analysis pipeline, which identified SETD2 as a radiosensitivity signature. Multi-omics analysis revealed enhanced chromatin accessibility and gene transcription by SETD2. In both LUAD bulk RNA sequencing (RNA-seq) and single-cell RNA sequencing (scRNA-seq), we found that SETD2-associated positive transcription patterns were associated with DNA damage responses. SETD2 knockdown significantly upregulated tumor cell apoptosis, attenuated proliferation and migration of LUAD tumor cells, and enhanced radiosensitivity *in vitro*. Moreover, SETD2 was a favorably prognostic factor whose effects were antagonized by the m6A-related genes RBM15 and YTHDF3 in LUAD. In brief, SETD2 was a promising epigenetic biomarker in LUAD patients.

## 1 Introduction

Lung cancer is one of the main causes of cancer-related deaths worldwide (Ferlay et al., 2019; Sung et al., 2021). Approximately 85% of lung cancers are non-small cell lung cancers (NSCLC), of which around half are lung adenocarcinomas (LUAD) (Behrend et al., 2021). The prognosis of lung cancer is still unsatisfactory (Goldstraw et al., 2016). Radiotherapy has clear benefits for patients unsuitable for surgery, and is widely used in the radical and palliative treatment of LUAD patients (Ettinger et al., 2021). Radioresistance is a major cause of lesion recurrence and metastasis (Pollom et al., 2016). In the era of precision medicine, research is gradually shifting from the population level to the individual level. Radiosensitivity is not only determined by tumor histology, but also affected by gene pathways (Césaire et al., 2022).

With the development of sequencing technology, a large amount of omics sequencing data have been documented (Hanash et al., 2002). Multi-omics data provided unparalleled dimensions of information and reflected the inherent features of individuals (Xu et al., 2021). Previous studies have successfully developed a genome-based model for adjusting radiotherapy dose (GARD) (Scott et al., 2017). Therefore, the identification of potential biomarkers is conducive to the development of precision radiotherapy.

Epigenetic regulation affects tumor heterogeneity, and is involved in radiosensitivity (Peng et al., 2021). On the post-transcriptional level, N6-methyladenosine (m6A) modification transferase METTL3 increases radioresistance *via* promoting the stability of target RNAs in multiple cancers (Huang et al., 2021). For histone modifications, histone deacetylase (HDAC) inhibitors demonstrate radiosensitization of various cancers in preclinical studies *via* targeting DNA damage responses (DDR) (Shirbhate et al., 2020). Moreover, Xue et al. (2015); Wu et al. (2020) found upregulated DNA methyl-transferase DNMT3B in radioresistant nasopharyngeal and prostate cancer cells. However, the impact of epigenetics on radiosensitivity is still not well understood, and the identification of novel epigenetic markers has a substantial clinical interest.

SETD2 is the sole transferase of histone H3 trimethylation on lysine 36 (H3K36me3) in humans. SETD2 is involved in DNA repair and maintaining chromatin integrity (Carvalho et al., 2014; Pfister et al., 2014). SETD2 is necessary to recruit DDR factors 53BP1 and RAD51 (Carvalho et al., 2014). In the Mayo cohort, renal carcinoma patients without H3K36me3 had worse cancer-specific survival (Ho et al., 2016). Moreover, SETD2 mutation promotes MLL-AF9-induced leukemia progression and chemoresistance (Mar et al., 2017). Our previous studies found that SETD2 knockdown triggers DNA double-strand breaks (DSB) and activates the cGAS-STING pathway (Zeng et al., Forthcoming 2022). On the other hand, SETD2-mediated H3K36me3 guides m6A modifications on nascent RNA transcripts (Kumari and Muthusamy, 2020). However, studies on the roles of SETD2 in LUAD are still lacking. Therapeutical values and possible mechanisms of SETD2 remain to be investigated in LUAD.

Here, we utilized comprehensive omics-data analysis to determine that SETD2 was a key radiosensitivity-related signature. Our results indicated that SETD2 enhanced chromatin opening and transcription, especially in the DDR-related pathways. *In vitro* experiments indicated that SETD2 knockdown upregulated tumor cell apoptosis, attenuated proliferation and migration of LUAD cells, and enhanced their radiosensitivity. Furthermore, SETD2 was a prognostic protection factor whose effect interacted with m6A-related genes. Our finding suggested SETD2 as a potential epigenetic marker in LUAD patients.

## 2 Materials and methods

### 2.1 Collection and processing of omics data

A total of 11 datasets were included in this study (Supplementary Table S1). The survival fraction at 2 Gy (SF2) was a common index to describe cellular radiosensitivity. In this study, the determination of SF2 was based on previous studies, which reported colony formation with irradiation (Supplementary Table S2) (Torres-Roca et al., 2005; Eschrich et al., 2009; Gao et al., 2012; Oleinick et al., 2016; Zhong et al., 2016). For Microarray data, GEO datasets were standardized by the default method. When multiple probes corresponded to the same gene, the maximum value of the probes was selected. GSE20549 contained 42 samples of H460 and H1299 cells at six time points (0, 2, 4, 8, 12, and 24 h) after 2 Gy ionizing radiation (IR). We collected 10 NSCLC cell samples from GSE32036 and 16 samples from GSE57083 (Byers et al., 2013), which were normalized and integrated with Z-score. GSE5949 contained 59 pan-cancer cell samples (Reinhold et al., 2010). We collected SETD2 expression data for survival analysis in GSE50081 (Der et al., 2014) and GSE3141 (Bild et al., 2006). For RNA-seq data, we collected RNA-seq (standardized by RPKM) of the 16 HepG2 cell samples treated with shSETD2 from GSE121949 (Huang et al., 2019). The clinical data of The Cancer Genome Atlas (TCGA) cohorts were downloaded by R TCGAbiolinks package (Colaprico et al., 2016). Xena was used to obtain omics data of TCGA (Goldman et al., 2020). The RNA-seq data were normalized by log2 (TPM +1). ChIP-seq data was annotated by R ChIPseeker package (Yu et al., 2015). H3K36me3 ChIP-seq of HepG2 cells with/without shSETD2 was obtained from GSE110318 (Huang et al., 2019). The lung cancer ChIP-seq data (H3K36me3, H3K27me3, H3K9me3, H3K27ac, H3K4me3, and H3K4me1) were downloaded from Roadmap (ID: EN96) (Kundaje et al., 2015). Moreover, we collected scRNA-seq for 43,704 cells from tumor tissues of 11 LUAD patients in GSE131907 (Kim et al., 2020). ATAC-seq data of TCGA was obtained from NCI GDC (https://gdc.cancer.gov/about-data/publications/ATACseq-AWG). The R ChIPseeker package was also used for annotation (Yu et al., 2015). To compare the expression of SETD2 in different NSCLC cell lines, we collected SETD2 RNA-seq in the Cancer Cell Line Encyclopedia (CCLE) database (Barretina et al., 2012).

### 2.2 Cell culture and radiation

The Type Culture Center of the Chinese Academy of Sciences (Shanghai, China) provided the LUAD A549 and H1299 cells, cultivated in RPMI-1640 media (HyClone, United States) containing 10% fetal bovine serum. The cells were grown in a standard tissue culture incubator at 37°C, with 95% humidity and 5% CO_2_. Radiation was conducted using a small animal radiation research platform (6 Gy, PXI X-RAD 225Cx, Gulmay, CT, United States).

### 2.3 Cell transfection

Small interfering RNAs (siRNAs) and negative control (NC) were transfected at 20 nM *via* jetPRIME® transfection reagent. SETD2 siRNA (siSETD2) 2 sequences were as follows: sense, CCU​UCA​GGC​UCA​GAG​UUA​ATT, and anti-sense, UUA​ACU​CUG​AGC​CUG​AAG​GTT; siSETD2 3 sequences were as follows: sense, CCG​GAA​ACC​UGA​CUG​CAA​ATT, and anti-sense, UUU​GCA​GUC​AGG​UUU​CCG​GTT.

### 2.4 RNA isolation and quantitative real-time PCR

Using the TRIzol reagent, total RNA was isolated from cells (Vazyme, China). We used HiScript® Q RT SuperMix (Vazyme, China) to transcribe RNA and ChamQTM SYBR® qPCR Master Mix (Vazyme, China) for qRT-PCR. The relative mRNA levels were calculated with the 2^−ΔΔCt^ method. All experiments were performed in triplicates.

### 2.5 Wound healing and colony formation assays

For wound healing assays, we seeded the transfected cells into 6-well plates. A straight line was scratched with a pipette tip. The migration rate was calculated using the following formula: wound closure rate (%) = (area of initial scratch—the area of final imaged cell-free area)/area of initial scratch * 100. For colony formation assays, we subjected the transfected cells to radiotherapy, and they were seeded into 6-well plates at 1,000 cells/well 48 h later. After 2 weeks, the medium was aspirated, and 4% paraformaldehyde was added and fixed for 30 min. Then after PBS washing, they were stained with 0/5% crystal violet for 30 min and finally washed with water, dried, and photographed.

### 2.6 Flow cytometry for cell apoptosis

After 48 h, the treated cells were collected and washed twice with PBS. We suspended the cells in binding buffer with Annexin V-FITC staining solution and propidium iodide (PI) solution on ice. The samples were detected by flow cytometry (Beckman, China).

### 2.7 Immunohistochemistry from the human protein atlas database

We collected SETD2 immunohistochemistry images from the HPA database (https://www.proteinatlas.org/) (Karlsson and Zhang, 2021), including available 5 LUAD and 3 normal lung tissues. All images were made of antibody HPA042451. SETD2 staining score was calculated as intensity times quantity. The intensity score consisted of 0 (Negative), 1 (Weak), 2 (Moderate), and 3 (Strong). The quantity score consisted of 0 (None), 1 (<25% cells), 2 (25–75% cells), and 3 (>75% cells).

### 2.8 Analysis of single-cell RNA sequencing

The Seurat workflow was adopted to analyze scRNA-seq data (Satija et al., 2015). Cells with less than 200 genes (min.features = 200) and genes with less than 3 cells (min.cells = 3) were screened out. Only the cells with less than 15% of mitochondrial genes were retained. A total of 2,000 hypervariable genes were selected with the vst method. An Elbow diagram was drawn to select the best number of principal components. The resolution parameter was set as 0.5. Uniform manifold approximation and projection were used to visualize single-cell atlas (McInnes et al., 2018), which was realized by Seurat DimPlot and FeaturePlot functions. Cell types were identified using marker genes from the previous study (Lambrechts et al., 2018). Specifically, tumor cell markers were EPCAM and KRT19; T/NK cell markers were NKG7, CD3E, CD3G, and CD3D; B cell markers were CD79A and CD79B; myeloid cell marker was LYZ; mast cell markers were TPSB2 and TPSAB1; fibroblast markers were COL1A1 and COL1A2; endothelial cell marker was CLDN5; normal epithelial cell marker was CAPS. GSVA was used to calculate the gene set enrichment score of individual cells (Hanzelmann et al., 2013).

### 2.9 Principal component analysis

PCA was a classic linearly dimensionality reduction algorithm. We used the R FactoMineR package to perform PCA (Lê et al., 2008). The first principal component was considered to be the vector with the largest variance. In this study, since gene clusters contained a large number of genes, we used the first principal component as the eigenvalue to characterize the gene clusters.

### 2.10 Short time-series expression miner analysis

Short time-series expression miner analysis was an algorithm to cluster, compare and visualize time-course gene expression (Ernst and Bar-Joseph, 2006). Genes with similar time expression patterns were grouped into the same clusters. We extracted eigenvalue of time-course clusters using PCA. Next, we performed Spearman’s correlation between the eigenvalue of gene clusters and SF2 to recognize SF2-related clusters.

### 2.11 Random forest

The RF was the ensemble methods with multiple decision trees. We used R randomForest packages to train RF model for SF2 fitting (Breiman, 2001). The mean-square error was used to calculate importance of genes.

### 2.12 Single-gene liner quadratic model

The Linear-Quadrac (LQ) model proposed by Kellerer and Rossi was a classical model widely used in the field of radiotherapy (Kellerer and Rossi, 1978). The LQ model estimated the survival fraction (SF) of cells exposed to radiation:

$$\text{SF}=e^{-\alpha \times D-\beta \times D^{2}}$$

Which e represents the natural logarithm, α and β represent radiation-specific parameters describing the radiosensitivity of tumor cells, D is the radiation dose.

However, the equation for fitting SF using gene expression was still unclear. We next analyzed a gene expression data set exposed to different doses (0, 2, 5, 6, and 7 Gy) of radiation (GSE102971, *n* = 100) (Park et al., 2017). Compared with quadratic equation and cubic equation, linear equation (dose-gene expression) has the smallest Akaike information criterion in the analysis of each gene, suggesting that gene expression was linearly related to radiation dose (Supplementary Figure S1). In order to establish a simulation model of gene expression and SF, we constructed a single-gene linear-quadratic (SGLQ) model inspired by the LQ model:

$$\text{SF}=e^{\alpha \times Ei-\beta \times Ei^{2}}$$

Here, α is the linear radiosensitivity parameter of a single gene i, and β represents the quadratic radiosensitivity parameter of gene i. Ei is the expression value of gene i. The SGLQ contributed to modeling the relationship between gene expression and SF in the era of omics.

### 2.13 The 4-omics system biological network

Gene regulations were the complex systems biology networks. Analysis of nodes in the network helped to identify key genes. The 4-omics system biological networks consisted of mutation, copy number alteration (CNA), mRNA co-expression and protein interaction sub-networks. The gene interaction of protein interaction sub-network was formed by STRING database (Szklarczyk et al., 2017). The mutation and CNA sub-networks were constructed by HotNet diffusion-oriented subnetworks (HotNet2) algorithm, which was based on random walk with restart (Leiserson et al., 2015). The HotNet2 included not only the topology of gene interaction networks from STRING, but also the heat values. Here, mutation frequency and copy number were set as heat values of the mutation and CNA sub-networks. Finally, HotNet2 identified sub-networks with close topology structure and high overall thermal diffusivity. The mRNA co-expression sub-network was formed by weight gene co-expression analysis (WGCNA) (Langfelder and Horvath, 2008). In this study, we used multi-omics data in TCGA pan-cancer cohorts to build the 4-omics system biological networks. Network analysis and visualization were realized by Cytoscape (Shannon et al., 2003). The innovation of this network was the inclusion of multi-omics data.

### 2.14 The maSigPro algorithm

Differential expression analysis of time-course transcriptome was performed by maSigPro using a 2-step regression strategy (Conesa et al., 2006). Since GSE121949 contained gene expression data at 4 time points (0, 1, 3, and 6 h) (Huang et al., 2019), we constructed the cubic equation in the maSigPro algorithm to identify treatment group related genes.

### 2.15 Binding and expression target analysis

BETA was a tool to integrate ChIP-seq and gene differential expression list from transcriptome (Wang et al., 2013). In this study, we explored the transcriptional activation or inhibition of H3K36me3, and identified the motif of H3K36me3 and its collaborators by combining H3K36me3 ChIP-seq (GSE110318) and RNA-seq (GSE121949) *via* BETA (Huang et al., 2019). BETA was realized by cistrome (http://cistrome.org/ap/root) (Liu et al., 2011).

### 2.16 Enrichment analysis

Gene set enrichment analysis (GSEA) was used to identify GO terms that were activated or inhibited in a predefined list of gene differential expression *via* permutation test (Ashburner et al., 2000). Over-representation analysis (ORA) was performed to identify GO terms associated with a predefined gene set *via* a hypergeometric test. GSEA and ORA were realized by R clusterProfiler packages (Yu et al., 2012).

### 2.17 Weighted gene co-expression network analysis

WGCNA clustered genes into different modules according to expression similarity through kmeans clustering and dynamic branch cutting (Langfelder and Horvath, 2008). In this study, we correlated the eigenvalues of the modules with the SETD2 expression to identify SETD2-related modules.

### 2.18 Statistical analysis

Most statistical analysis was analyzed in R software 4.1.0. The basic statistical analysis was performed by the R stats package. Cox proportional hazards regression was realized by the R survival package. When the survival curve crossed, landmark analysis was used to assess the prognostic value of SETD2 at different time periods (Der et al., 2014). The landmark analysis was realized by R jskm package (https://rdrr.io/cran/jskm/). Gene expression plots for TCGA data were implemented by GEPIA (Tang et al., 2017a) and TIMER (Li et al., 2017). *p* values less than 0.05 were considered statistically significant. All the *p* values were two-sided.

## 3 Results

### 3.1 Comprehensive analysis suggested a critical role of SETD2 in radiosensitivity

#### 3.1.1 Identification of radiosensitivity related transcriptome patterns

Gene expression induced by temporal changes in radiation may be related to radiotherapy response and sensitivity. We collected 42 NSCLC cell samples from GSE20549 (Clough and Barrett, 2016) with six time points (0, 2, 4, 8, 12, and 24 h) after 2 Gy IR. The ANOVA identified 3,337 genes variously expressed at different time points (*p* < 0.05). We next explored IR time-dependent gene patterns using Short Time-series Expression Miner method (Ernst and Bar-Joseph, 2006). A total of 11 time-course gene clusters reached statistical significance (false discovery rate, FDR q < 0.01, Supplementary Figure S2).

**FIGURE 1:**
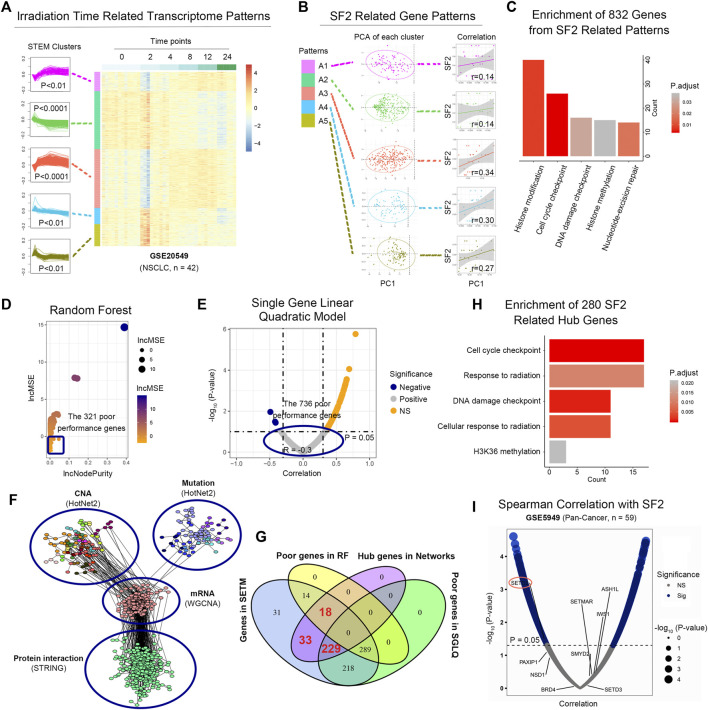
SETD2 functions as a radiosensitivity signature. **(A)** Radiosensitivity-related time-course clusters by the short time-series expression miner method. **(B)** Correlation between SF2 and the first principal component (PC1) of each time-course cluster. **(C)** GO enrichment analysis of the 832 genes in 5 SF2-related clusters. **(D)** RF analysis of single gene on SF2 in GSE32036 and GSE57083. **(E)** SGLQ analysis of single gene on SF2 in GSE32036 and GSE57083. **(F)** The 4-omics biological networks included mutation, CNA, mRNA co-expression, and protein interaction sub-networks. **(G)** Venn diagram for screening key genes. **(H)** GO enrichment analysis of the 280 hub genes. **(I)** Correlation between gene expression and SF2.

To determine the radiosensitivity-related time-course clusters, we collected 26 untreated NSCLC cell samples from GSE32036 and GSE57083 (Byers et al., 2013), whose SF2 were provided by colony formation assays from previous studies (Supplementary Table S2, see Methods). There were 5 first principal component of clusters correlated with SF2 (cor >0.1, Figures 1A,B), which were considered as SF2-related clusters. Gene ontology (GO) enrichment analysis suggested that the 832 genes in these 5 clusters were linked to cell cycle, DDR and histone methylation (all, FDR *p* < 0.05, Figure 1C).

#### 3.1.2 SETD2 was a radiosensitivity signature at the single-gene scale

Since the above analysis was performed at the gene cluster level, we subsequently determined the SF2-related signatures at the single-gene level. Here we used the RF (Breiman, 2001) and SGLQ model to quantitate the importance of a single gene on SF2 in GSE32036 and GSE57083. A total of 289 (34.7%) genes did not perform well both in the RF and SGLQ models (Figures 1D,E), suggesting that these genes were unlikely to be SF2-related signatures.

In the remaining 543 (65.3%) genes, we next identified the hub ones. We constructed the “4-level network” (see Methods), containing the mutation, CNA, RNA, and protein subnetworks from TCGA pan-cancer data (Figure 1F). The 3 subnetworks were identified in the 4-level biological network, representing histone methylation, cell cycle, and DNA damage checkpoint (Supplementary Figure S3). Considering the topological structure of networks, the 280 genes were selected as irradiation-related genes (Figure 1G) with a high degree, betweenness, and closeness centrality (all, > median value). The significant enrichment of histone H3K36 methylation, cell cycle, DDR were observed in these 280 genes (Figure 1H), containing ATM, ERCC4, H2AFX, DTX3L, CCNA2, RAD9A, POLE3, BRSK1, CLOCK, CNOT3, CNOT4, CNOT6, BRD4, CAMK2A, EZH2, RB1, ACTR1A, AURKB, RBX1, NSD1.

We next validated the relationship between H3K36 methylation regulatory genes (SETD2, SETD3, NSD1, PAXIP1, BRD4, IWS1, SETMAR, SMYD2, ASH1L) and SF2 in the dataset GSE5949 (pan-cancer cell lines, *n* = 59) (Reinhold et al., 2010). SETD2 was the only gene linked to SF2 *via* Spearman correlation analysis (*p* = 0.04, Figure 1I).

### 3.2 SETD2 enhanced transcription and chromosomal accessibility

**FIGURE 2:**
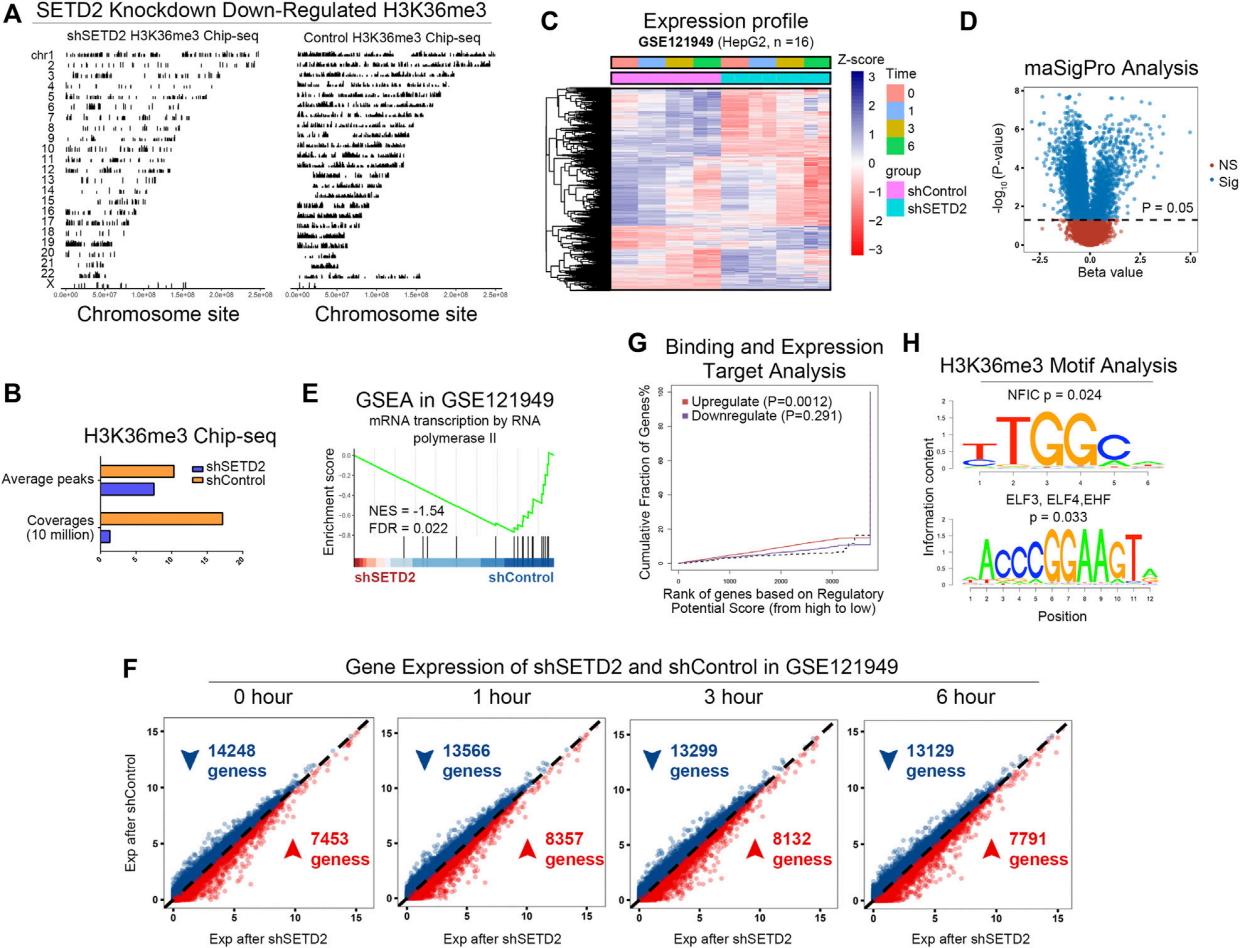
SETD2 and H3K36me3 enhanced transcription. **(A)** The H3K36me3 signals of shSETD2 vs. control in GSE110318. **(B)** The quantitative H3K36me3 signals. **(C)** Expression profile of GSE121949. **(D)** Gene different expression analysis between shSETD2 and control groups using maSigPro algorithm. **(E)** GSEA of shSETD2 vs. control in GSE121949. **(F)** Gene expression of shSETD2 vs. control in GSE121949. **(G)** Binding and expression target analysis (BETA) of H3K36me3 Chip-seq (GSE110318) and RNA-seq (GSE121949). **(H)** Motif analysis of H3K36me3 Chip-seq.

SETD2 was the main methyltransferase that specifically trimethylated “Lys-36” of histone H3 in mammals. The H3K36me3 signals decreased in the whole genome after SETD2 knockdown in GSE110318 (Huang et al., 2019) (Figure 2A), which affected both H3K36me3 coverage and average peak signals (Figure 2B).

GSE121949 (Huang et al., 2019) provided RNA-seq of HepG2 cells with or without shSETD2 at 4 time points (0, 1, 3, and 6 h, Figure 2C). We performed gene differential expression analysis between the shSETD2 and control groups using the maSigPro algorithm (Conesa et al., 2006) (Figure 2D). Enrichment analysis showed inhibited transcriptiona in the shSETD2 groups (Figure 2E). Compare with the control group, more genes were downregulated in the shSETD2 group at all time points (Figure 2F). Furthermore, binding and expression target analysis (BETA) (Wang et al., 2013) of integrating H3K36me3 ChIP-seq (GSE110318) and RNA-seq (GSE121949) demonstrated that H3K36me3 enhanced transcription (Figure 2G). Motif analysis of H3K36me3 ChIP-seq suggested that H3K36me3 regulated transcription factors (NFIC, ELF2, ELF4, EHF, Figure 2H).

**FIGURE 3:**
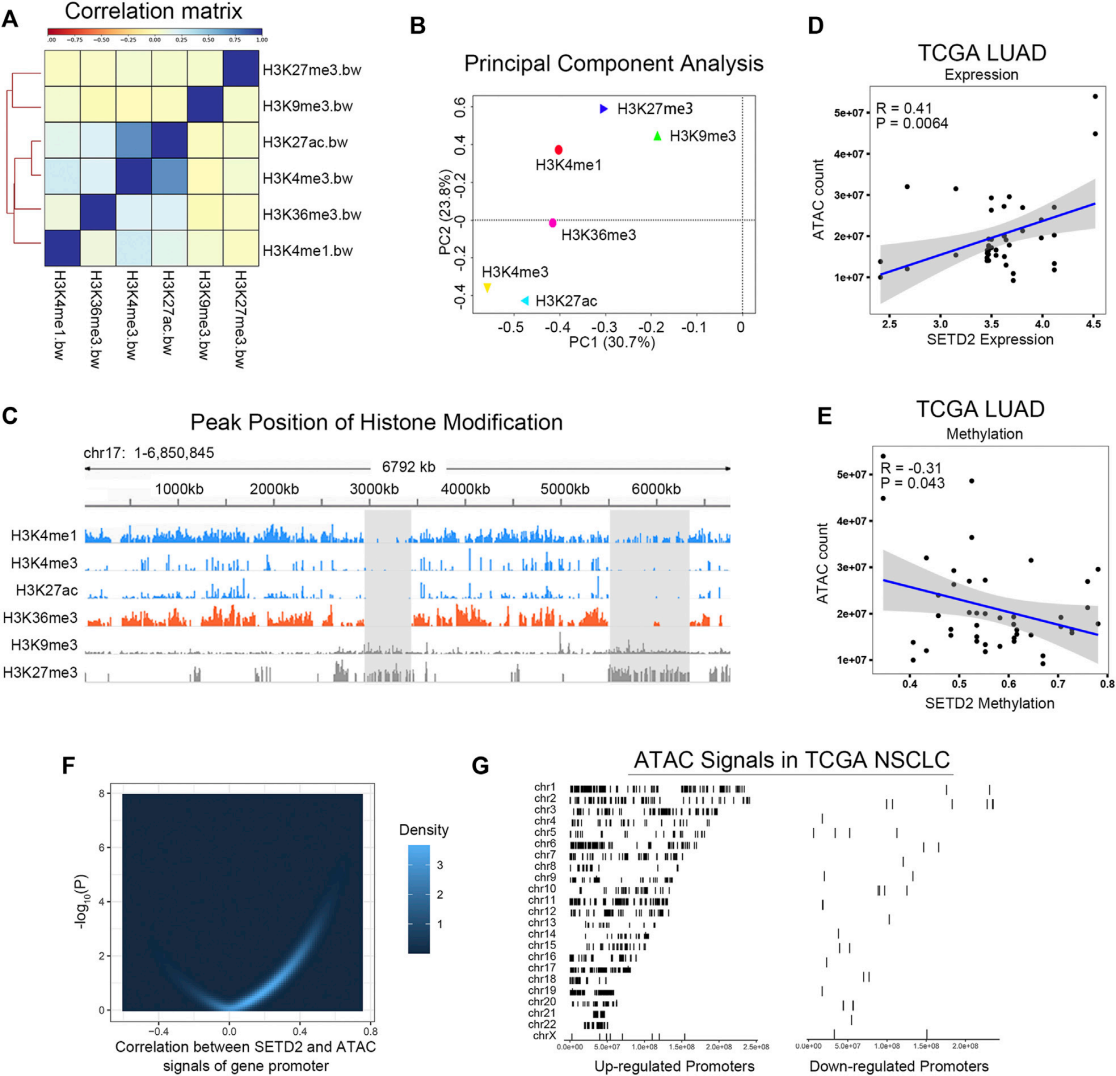
SETD2 and H3K36me3 enhanced chromosomal accessibility. **(A)** Correlation analysis of H3K36me3 and other histone modifications. **(B)** PCA of H3K36me3 and other histone modifications. **(C)** Peak distributions in chromosome 17: 1-6850845 of H3K36me3 and other histone modifications. **(D,E)** Correlation between SETD2 and ATAC signals in TCGA LUAD. **(F)** Correlation between SETD2 expression and the accessibility of the whole gene promoters. **(G)** The left side shows the location of the ATAC-seq signal of genes whose promoter region accessibility is positively correlated with SETD2 on the chromosome; The right side shows the position of the ATAC-seq signal of the negatively related gene on the chromosome.

Open chromatin facilitates transcription. Next, we investigated the relation between SETD2/H3K36me3 and chromosomal accessibility. We collected ChIP-seq data for the six histone modifications (H3K36me3, H3K27me3, H3K9me3, H3K27ac, H3K4me3, and H3K4me1) of lung cancer sample from Roadmap (Sample ID: EN96) (Kundaje et al., 2015). Correlation analysis and PCA showed that H3K36me3 had the similar patterns to open-chromosome-related histone modifications (H3K27ac, H3K4me3), but was distant from closed histone modifications (H3K27me3, H3K9me3, Figures 3A,B). Figure 3C showed a specific example of peak distributions in chromosome 17: 1-6850845. Furthermore, ATAC-seq of TCGA cohorts demonstrated that expression and promoter methylation of SETD2 were associated with chromosomal accessibility in TCGA LUAD (Figures 3D,E). The combined analysis of ATAC-seq and RNA-seq showed that the expression of SETD2 was positively correlated with the openness of the promoter region of the extensive genes in TCGA NSCLC (*n* = 76, Figures 3F,G). These results indicated that SETD2 enhanced transcription and chromosomal accessibility.

### 3.3 SETD2 positively regulated transcriptional patterns associated with DNA damage responses

**FIGURE 4:**
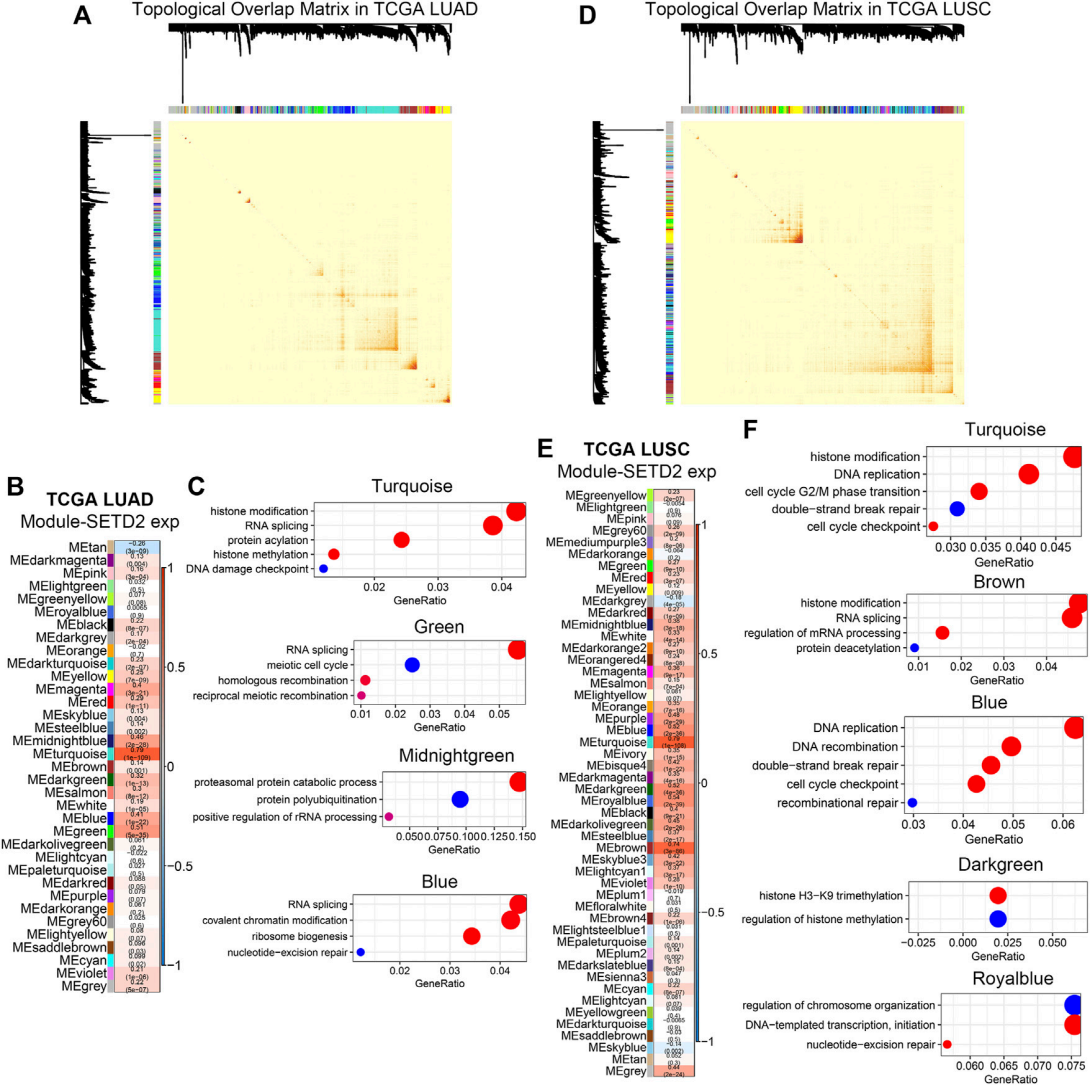
SETD2 regulated transcriptional patterns in bulk RNA-seq. **(A)** Visualization of topological overlap matrix in TCGA LUAD. **(B)** WGCNA revealed the gene clusters related to SETD2 in TCGA LUAD. **(C)** GO enrichment analysis of SETD2-related clusters in TCGA LUAD. **(D)** Visualization of topological overlap matrix in TCGA LUSC. **(E)** WGCNA revealed the gene clusters related to SETD2 in TCGA LUSC. **(F)** GO enrichment analysis of SETD2-related clusters in TCGA LUSC.

The next question was whether the enhanced transcription by SETD2 was gene-specific. We analyzed SETD2-related co-expression genes in bulk RNA-seq. In the TCGA LUAD cohort, WGCNA (Langfelder and Horvath, 2008) identified 35 co-expression modules (Figure 4A, Supplementary Figures S4, S5). Correlation analysis of module eigenvalues with SETD2 expression revealed the 4 SETD2 positive correlation modules (Turquoise, Green, Midnightgreen, and Blue modules, Figure 4B). GO enrichment analysis showed that genes of the above 4 modules enriched in DDR, DNA repair, RNA splicing, and histone modification signals (Figure 4C).

In the TCGA lung squamous cell carcinoma (LUSC) cohort, we repeated the WGCNA (Figure 4D, Supplementary Figures S6, S7). Similarly, we identified 50 modules, the 5 of which were positively correlated with SETD2 (Turquoise, Brown, Blue, Darkgreen, and Royalblue Figure 4E). DNA damage repair, cell cycle, RNA splicing, and histone modification signals were enriched in genes of these 5 modules (Figure 4F).

### 3.4 Single-cell analysis validated the co-expression patterns of SETD2 in lung adenocarcinoma

**FIGURE 5:**
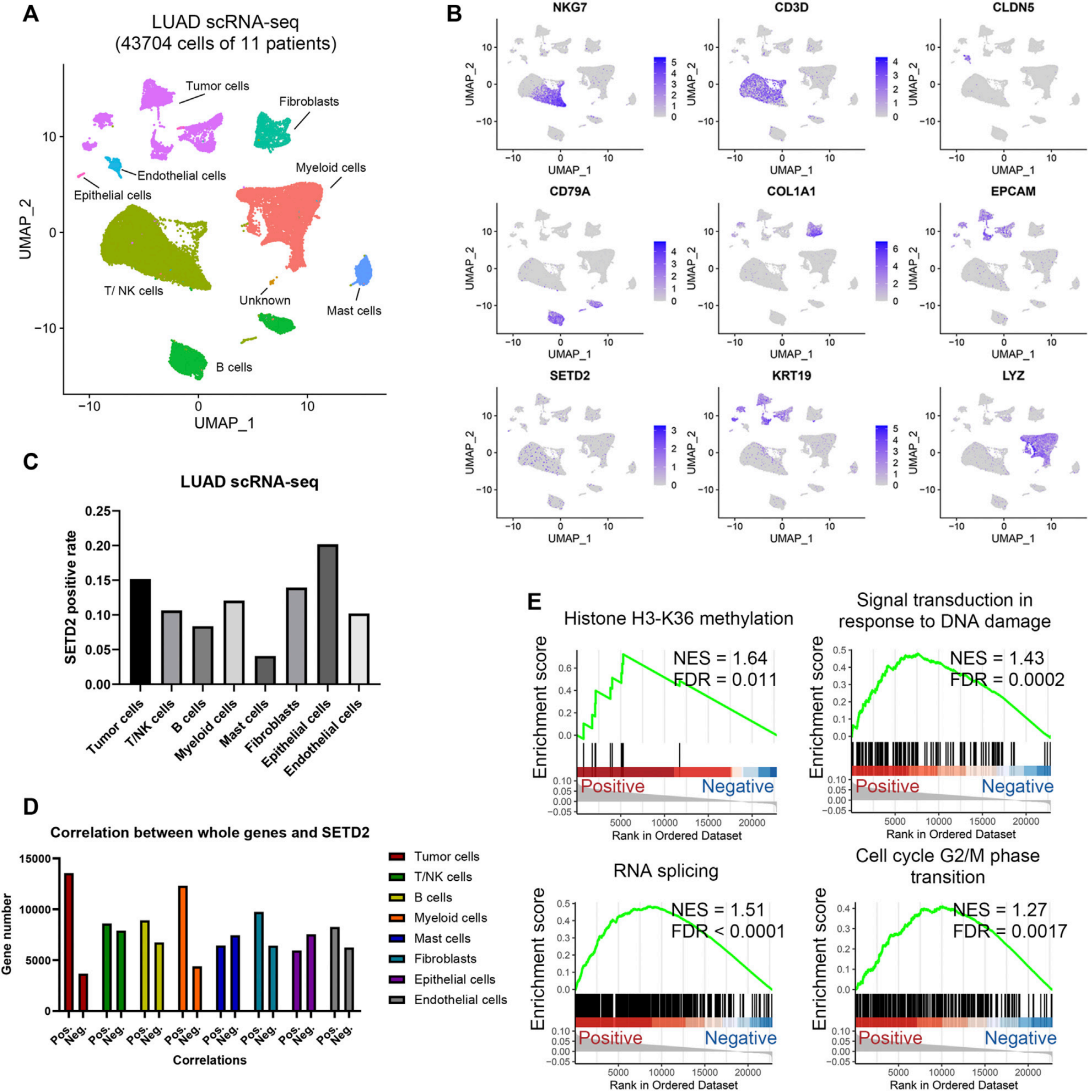
Analysis of SETD2 in LUAD scRNA-seq. **(A)** UMAP of the 43,704 cells in LUAD scRNA-seq. **(B)** Marker gene expressions in scRNA-seq. **(C)** Positive rate of SETD2 for each cell type. **(D)** The number of genes positively or negatively correlated with SETD2 expression. **(E)** GSEA of positive SETD2 vs. negative cells in scRNA-seq.

We next investigated 43,704 single-cell RNA profiles of the 11 primary LUAD patients in scRNA-seq GSE131907 (Kim et al., 2020). Through Seurat workflows, we identified the 8 cell types: tumor cells, fibroblasts, endothelial cells, epithelial cells, T/NK cells, B cells, myeloid cells, and mast cells (Figure 5A). SETD2 was widely distributed in different cell types (Figure 5B, Supplementary Figure S8). Overall, The SETD2 positive rate in tumor cells was lower than that in normal epithelial cells (15.2 vs. 20.2%, *p* = 0.2, Figure 5C), but higher than that in immune cells, including T/NK cells (10.7%, *p* < 0.0001), B cells (8.4%, *p* < 0.0001), and myeloid cells (12.1%, *p* < 0.0001). Moreover, in log2 (TPM+1) normalized profiles, we found that SETD2 was positively associated with the expression of more genes (Figure 5D), especially in tumor cells (positive rate: 78.7%) and myeloid cells (positive rate: 73.7%), while the opposite was observed in mast cells (positive rate: 46.4%) and epithelial cells (positive rate: 44.1%). This finding was consistent with Section 3.2. Next, we compared the gene expression of SETD2-positive and negative tumor cells. Genes highly expressed in SETD2-positive tumor cells were enriched in DDR, RNA splicing, and histone modification signals (Figure 5E).

### 3.5 Knockdown of SETD2 upregulated apoptosis, attenuated proliferation and migration of tumor cells, and enhanced the radiosensitivity in lung adenocarcinoma

SETD2 was downregulated in TCGA LUAD and LUSC tissues than normal ones (Supplementary Figure S9). In immunohistochemistry of 5 LUAD and 3 normal lung tissues from the HPA database (Karlsson and Zhang, 2021), we compared SETD2 staining scores of tumor and alveolar cells. The results showed that SETD2 staining scores were low but not significantly different between tumor and alveolar cells, and that the staining was mainly concentrated in the nuclear (Supplementary Figures S10A,B).

**FIGURE 6:**
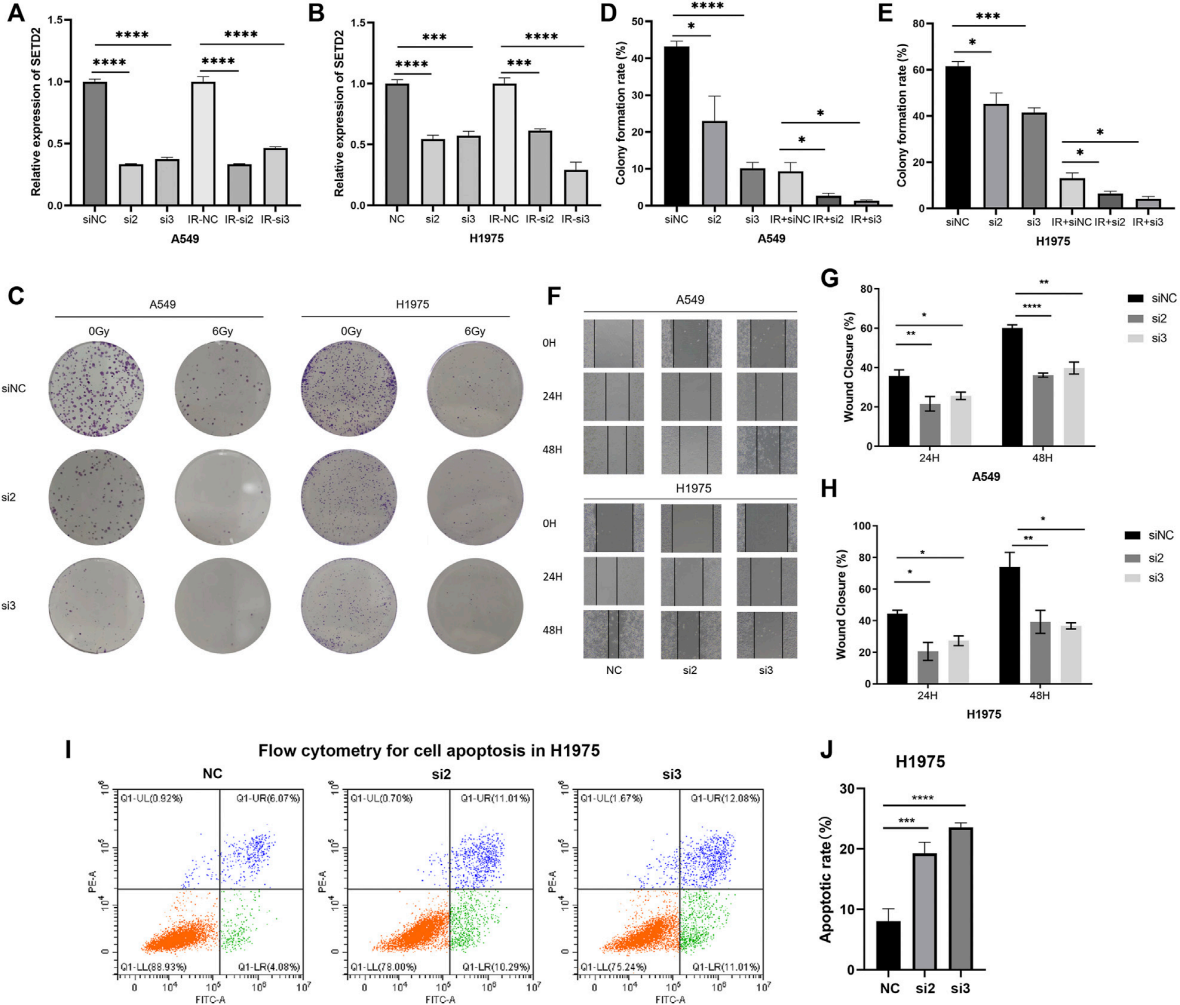
Flow cytometry, wound healing assays and clone formation experiments treated with siSETD2 or radiation. **(A)** The qRT-PCR of SETD2 in A549 cells. **(B)** The qRT-PCR of SETD2 in H1975 cells. **(C)** The six well plate image of clone formation experiment. **(D)** Clone formation rate of A549 cells. **(E)** Clone formation rate of H1975 cells. **(F)** Wound healing assay image. **(G)** Wound closure rate of A549 cells. **(H)** Wound closure rate of H1975 cells. **(I)** Flow cytometry for cell apoptosis in H1975. **(J)** Quantitative results of apoptosis.

We next investigated the effects of SETD2 on tumor malignant behaviors and radiosensitivity *in vitro*. According to our preliminary studies (Zeng et al., Forthcoming 2022), the expression levels of SETD2 in H1975 and A549 cells were high. In this study, we collected RNA-seq for NSCLC cells in the CCLE dataset. SETD2 expression remained higher in H1975 and A549 cells than H1299, PC9, and H460 cells (Supplementary Figure S11). Therefore, we cultured LUAD A549 and H1975 cells and divided them into six groups: negative control, siNC; siSETD2-2, si2; siSETD2-3, si3; negative control plus 6 Gy IR, IR-NC; siSETD2-2 plus 6 Gy IR, IR-s2; siSETD2-3 plus 6 Gy IR, IR-s3. With or without IR, siSETD2 showed high knockdown efficiency in A549 and H1975 (Figures 6A,B). Colony formation assays indicated attenuated tumor proliferation after SETD2 knockdown (Figures 6C–E). Cell proliferation was diminished after 6 Gy IR, and cells were more sensitive to radiation upon siSETD2 treatment. Moreover, SETD2 knockdown decreased cell migration (Figures 6F–H). Due to the severe killing of tumor cells by IR after transfection with siSETD2, we did not perform wound healing assays in the IR groups. Next, we performed flow cytometry for cell apoptosis in H1975 cells. SETD2 knockdown significantly upregulated LUAD cell apoptosis (Figures 6I,J).

### 3.6 SETD2 interacted with N6-methyladenosine-related genes RBM15 & YTHDF3 statistically and was associated with a favorable prognosis

**FIGURE 7:**
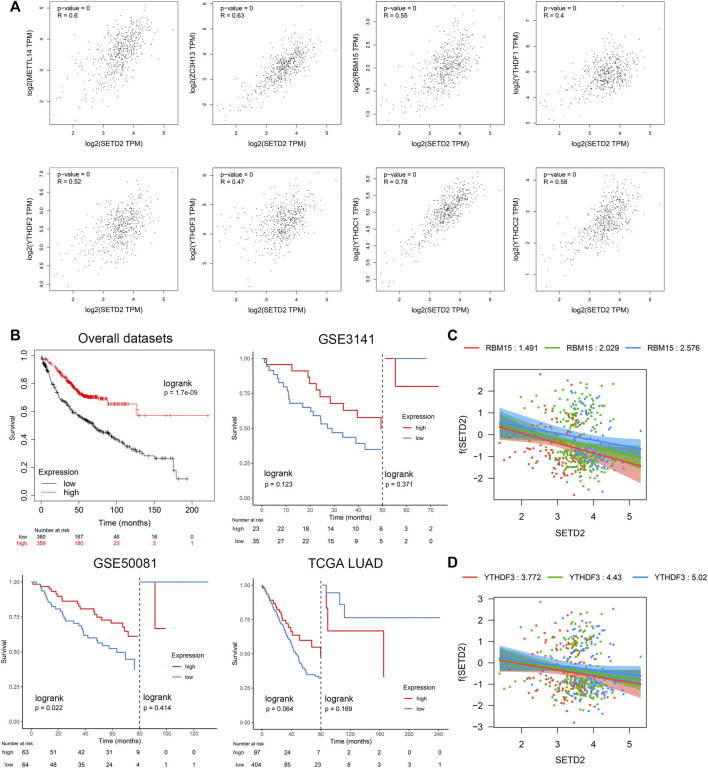
SETD2 was linked to favorable prognosis whose effect was negatively affected by the interaction of m6A-related genes RBM15 and YTHDF3. **(A)** Correlations between SETD2 and m6A-related genes. **(B)** Kaplan-Meier survival curves of SETD2 in the Kmplot database. **(C)** Visualization of interaction effect of RBM15 on Cox regression coefficient of SETD2. **(D)** Visualization of interaction effect of YTHDF3 on Cox regression coefficient of SETD2.

Previous studies reported the possible association between SETD2 and m6A (Kumari and Muthusamy, 2020). We comprehensively analyzed m6A-related genes, including 8 “writer” genes, 9 “reader” genes, and 2 “eraser” genes (Gu et al., 2020) in TCGA LUAD dataset. SETD2 was positively related to “writer” and “reader” genes (r > 0.4 & *p* < 0.01): METTL14, ZC3H13, RBM15, YTHDF1, YTHDF2, YTHDF3, YTHDC1, and YTHDC2 (Figure 7A, Supplementary Figure S12). SETD2 was linked to a favorable prognosis in multiple LUAD datasets (Figure 7B). However, the landmark analysis showed that patients with high SETD2 expression changed from favorable prognosis to unfavorable prognosis after more than 50–80 months. We next investigated the interaction effects of m6A-related genes with SETD2 on prognosis. Using multivariate Cox regression with interaction terms (Survival ∼ SETD2 + SETD2 *m6A gene + age + gender + stage), we identified that RBM15 (interaction term HR = 1.15, *p* = 0.02) and YTHDF3 (interaction term HR = 1.06, *p* = 0.18) interacted with SETD2 (Supplementary Tables S3, S4). The protective effects of SETD2 on prognosis were enhanced with the reduction of RBM15 or YTHDF3 (Figures 7C,D). The prognostic effects of SETD2 may be explained with low microsatellite instability and frequency of mutations (Liu et al., 2015; Zeng et al., Forthcoming 2022).

## 4 Discussion

Our work demonstrated that SETD2 as a radiosensitivity signature positively regulated DDR-related transcriptional patterns. Possibly due to inhibition of DDR, SETD2 knockdown upregulated the radiosensitivity of LUAD cells. Clinically, SETD2 was a promising epigenetic biomarker for prognosis and radiotherapy in LUAD.

Previous studies identified radiosensitivity-related genes based on regression. Torres-Roca et al. (2015) identified 10 genes associated with SF2 by linear regression models (Eschrich et al., 2009). Tang et al. (2017b) identified 65 radiosensitivity-related genes by logistic regression models in soft tissue sarcoma. In this study, we implemented a novel gene identification pipeline. Through pattern analysis of radiation time-associated transcriptomes, machine learning algorithms, and 4-omics networks, we successfully identified SETD2 as a key gene for radiosensitivity, which was validated in other omics datasets and cell experiments. Our novel pipeline can provide a case for other gene identification related studies.

Poly (ADP-ribose) polymerase (PARP) inhibitors targeted DNA damage repair, induced further DNA damage, and had a synthetic lethal effect in DNA repair-deficient tumors (Slade, 2020). PARP inhibitors improved progression-free survival in recurrent ovarian cancer patients with BRCA1/2 mutation and platinum-sensitive by 13.6 months (Pujade-Lauraine et al., 2017). PARP inhibitors also had a higher response rate in BRCA mutant triple-negative breast cancer (Pahuja et al., 2014). In addition to BRCA, other DNA repair-related genes also affected PARP inhibitor responses, such as RAD51 (Liu et al., 2017). Due to the important roles of SETD2 in homologous recombination repair (Skucha et al., 2019), the application of PARP inhibitor in SETD2-deficient tumors may achieve favorable curative effects, which needed to be confirmed by further studies.

SETD2 also played important roles in m6A RNA modification (Huang et al., 2019), which was related to prognosis and radioresistance. Li et al. (2020) found that low expression of FTO and METTL14 and high expression of METTL3, HNRNPA2B1, and YTHDF3 were related to the poor prognosis of osteosarcoma. The m6A “writer” METTL3 was demonstrated to promote radioresistance in pancreatic cancer (Taketo et al., 2018), hypopharyngeal squamous cell carcinoma (Wu et al., 2021), and glioma stem-like cells (Visvanathan et al., 2018). Radiosensitization caused by SETD2 knockdown may be related to both DDR and m6A.

LUAD and LUSC were highly heterogeneous for radiotherapy. Previous studies found that LUSC shrunk faster than LUAD after stereotactic body radiotherapy (Miyakawa et al., 2013). However, the local control rates of LUAD were not inferior to LUSC (Miyakawa et al., 2013; Hörner-Rieber et al., 2017; McAleese et al., 2019; Katagiri and Jingu, 2021). Moreover, LUSC was more likely to relapse locally, while LUAD was more likely to metastasize after radiation (McAleese et al., 2019; Katagiri and Jingu, 2021). For the overall survival of LUAD and LUSC after radiotherapy, there was some seemingly contradictory evidence (Nakayama et al., 1997; Holgersson et al., 2011), possibly due to the heterogeneity of the included populations. Despite the controversy, based on current evidence, the radiocurability of LUAD was not inferior to that of LUSC.

In the bulk transcriptome, SETD2 was associated with a favorable prognosis. However, it was unclear whether the favorable prognosis of SETD2 in LUAD was determined by the tumor or microenvironmental cells. Previous studies found that SETD2 histological staining scores of tumor cells were linked to good survival in gastric cancer (Chen et al., 2018) and nonmetastatic clear-cell renal cell carcinoma (Liu et al., 2015), possibly because lack of SETD2 increased microsatellite instability and frequency of spontaneous mutations (Liu et al., 2015; Zeng et al., Forthcoming 2022). Moreover, we collected 7 LUAD single-cell-derived metastasis-associated genes (PRSS3, GPI, CCL20, KRT18, TCN1, SLCO1B3, and GNPNAT1) from a previous study (He et al., 2021). However, in GSE131907, the results of GSVA analysis showed no significant difference in the scores of 7 metastasis-associated genes between SETD2-positive and negative tumor cells (Supplementary Figure S13). We expected further clinical studies to investigate the prognostic implications of SETD2 in cancer.

There were still some open issues. One focus was to identify genomic signatures associated with radioresistance and explore their mechanisms. Although acquired small deletion mutations were suggested as possible causes of radioresistance (Kocakavuk et al., 2021), further studies were required to investigate the complexity of tumor heterogeneity. The landmark analysis showed that the protective effects of SETD2 were reversed at more than 50–80 months, however the mechanism was not clear. Moreover, SETD2 also plays important roles in RNA splicing (Bhattacharya et al., 2021). Other mechanisms by which SETD2 affected therapeutic effectiveness also remained to be investigated. In addition, preclinical studies investigating the toxicity and efficacy of interventions targeting SETD2 were lacking.

In this study, we mainly analyzed various genomics data supplemented by a limited amount of *in vitro* cell data. This study had some limits: 1) Since SETD2 expression was not high in various cells, we expected further studies to confirm the implications of SETD2 over-expression in LUAD; 2) The role of SETD2 regulated m6A in radiosensitivity remains unclear; 3) More clinical evidence was needed to use SETD2 as a prognosis and radiotherapy marker.

## 5 Conclusion

Our comprehensive analysis pipeline demonstrated that SETD2 was a key radiosensitivity signature. SETD2 enhanced chromatin accessibility and gene transcription which focused on DDR, DNA damage repair, and histone modification. Knockdown of SETD2 attenuated the proliferation and migration of LUAD cells, and enhanced cell apoptosis and radiosensitivity *in vitro*. Furthermore, SETD2 was a positively prognostic factor whose effects were negatively affected by the interaction of m6A-related genes RBM15 and YTHDF3.

## Data Availability

The original contributions presented in the study are included in the article/Supplementary Material, further inquiries can be directed to the corresponding authors.

## Supplementary material

SUPPLEMENTARY FIGURE S1 Akaike information criterion of linear equation, quadratic equation and cubic equation: Radiation dose ∼ gene expression. The slope of the red line is 1 and the intercept is 0.

SUPPLEMENTARY FIGURE S2 Time-dependent gene clusters *via* Short Time-series Expression Miner. The blue line shows the changes of genes in the cluster over time. The black line is the eigenvalue of the cluster. The P value of the first 11 clusters is less than 0.01.

SUPPLEMENTARY FIGURE S3 The subnetworks in the 4-level biological network. **(A)** Subnetwork structures which were identified by MCODE app in Cytoscape (Shannon et al., 2003); **(B)** GO enrichment analysis of genes in the corresponding left subnetworks.

SUPPLEMENTARY FIGURE S4 Selection of soft threshold in scale-free co-expression networks in TCGA LUAD.

SUPPLEMENTARY FIGURE S5 Co-expression modules in TCGA LUAD.

SUPPLEMENTARY FIGURE S6 Selection of soft threshold in scale-free co-expression networks in TCGA LUSC.

SUPPLEMENTARY FIGURE S7 Co-expression modules in TCGA LUSC.

SUPPLEMENTARY FIGURE S8 Positive proportion of SETD2 in different cells of the 11 patients in scRNA-seq GSE131907.

SUPPLEMENTARY FIGURE S9 Expression of SETD2 in TCGA pan-cancer datasets.

SUPPLEMENTARY FIGURE S10 Immunohistochemistry of SETD2 from the HPA database. **(A)** SETD2 staining scores of LUAD tumor cells and alveolar cells. **(B)** The immunohistochemistry images of 5 lung adenocarcinoma and 3 normal lung tissues.

SUPPLEMENTARY FIGURE S11 SETD2 expression of NSCLC cell lines from the CCLE database.

SUPPLEMENTARY FIGURE S12 Correlations between SETD2 and m6A-related genes.

SUPPLEMENTARY FIGURE S13 GSVA analysis of 7 LUAD single-cell-derived metastasis-associated genes (PRSS3, GPI, CCL20, KRT18, TCN1, SLCO1B3, and GNPNAT1) between SETD2-positive and negative tumor cells in GSE131907.

SUPPLEMENTARY TABLE S1 The basic information of included datasets.

SUPPLEMENTARY TABLE S2 SF2 values from previous laboratory studies.

SUPPLEMENTARY TABLE S3 Multivariate Cox regression with RBM15 * SETD2 interaction.

SUPPLEMENTARY TABLE S4 Multivariate Cox regression with YTHDF3 * SETD2 interaction.

## References

[B1] Ashburner M. Ball C. A. Blake J. A. Botstein D. Butler H. Cherry J. M. (2000). Gene ontology: tool for the unification of biology. Nat. Genet. 25 (1), 25–29. 10.1038/75556 10802651 PMC3037419

[B2] Barretina J. Caponigro G. Stransky N. Venkatesan K. Margolin A. A. Kim S. (2012). The Cancer Cell Line Encyclopedia enables predictive modelling of anticancer drug sensitivity. Nature 483 (7391), 603–607. 10.1038/nature11003 22460905 PMC3320027

[B3] Behrend S. J. Giotopoulou G. A. Spella M. Stathopoulos G. T. (2021). A role for club cells in smoking-associated lung adenocarcinoma. Eur. Respir. Rev. 30 (162), 210122. 10.1183/16000617.0122-2021 34670807 PMC9488964

[B4] Bhattacharya S. Levy M. J. Zhang N. Li H. Florens L. Washburn M. P. (2021). The methyltransferase SETD2 couples transcription and splicing by engaging mRNA processing factors through its SHI domain. Nat. Commun. 12 (1), 1443. 10.1038/s41467-021-21663-w 33664260 PMC7933334

[B5] Bild A. H. Yao G. Chang J. T. Wang Q. Potti A. Chasse D. (2006). Oncogenic pathway signatures in human cancers as a guide to targeted therapies. Nature 439 (7074), 353–357. 10.1038/nature04296 16273092

[B6] Breiman L. (2001). Random forests. Mach. Learn. 45 (1), 5–32. 10.1023/A:1010933404324

[B7] Byers L. A. Diao L. Wang J. Saintigny P. Girard L. Peyton M. (2013). An epithelial-mesenchymal transition gene signature predicts resistance to EGFR and PI3K inhibitors and identifies Axl as a therapeutic target for overcoming EGFR inhibitor resistance. Clin. Cancer Res. 19 (1), 279–290. 10.1158/1078-0432.ccr-12-1558 23091115 PMC3567921

[B8] Carvalho S. Vítor A. C. Sridhara S. C. Martins F. B. Raposo A. C. Desterro J. M. P. (2014). SETD2 is required for DNA double-strand break repair and activation of the p53-mediated checkpoint. eLife 3, e02482. 10.7554/eLife.02482 24843002 PMC4038841

[B9] Césaire M. Montanari J. Curcio H. Lerouge D. Gervais R. Demontrond P. (2022). Radioresistance of non-small cell lung cancers and therapeutic perspectives. Cancers 14 (12), 2829. 10.3390/cancers14122829 35740495 PMC9221493

[B10] Chen Z. Raghoonundun C. Chen W. Zhang Y. Tang W. Fan X. (2018). SETD2 indicates favourable prognosis in gastric cancer and suppresses cancer cell proliferation, migration, and invasion. Biochem. Biophys. Res. Commun. 498 (3), 579–585. 10.1016/j.bbrc.2018.03.022 29522714

[B11] Clough E. Barrett T. (2016). The gene expression omnibus database. Methods Mol. Biol. 1418, 93–110. 10.1007/978-1-4939-3578-9_5 27008011 PMC4944384

[B12] Colaprico A. Silva T. C. Olsen C. Garofano L. Cava C. Garolini D. (2016). TCGAbiolinks: an R/bioconductor package for integrative analysis of TCGA data. Nucleic Acids Res. 44 (8), e71. 10.1093/nar/gkv1507 26704973 PMC4856967

[B13] Conesa A. Nueda M. J. Ferrer A. Talón M. (2006). maSigPro: a method to identify significantly differential expression profiles in time-course microarray experiments. Bioinformatics 22 (9), 1096–1102. 10.1093/bioinformatics/btl056 16481333

[B14] Der S. D. Sykes J. Pintilie M. Zhu C. Q. Strumpf D. Liu N. (2014). Validation of a histology-independent prognostic gene signature for early-stage, non-small-cell lung cancer including stage IA patients. J. Thorac. Oncol. 9 (1), 59–64. 10.1097/jto.0000000000000042 24305008

[B15] Ernst J. Bar-Joseph Z. (2006). STEM: a tool for the analysis of short time series gene expression data. BMC Bioinforma. 7 (1), 191. 10.1186/1471-2105-7-191 PMC1456994 16597342

[B16] Eschrich S. Zhang H. Zhao H. Boulware D. Lee J. H. Bloom G. (2009). Systems biology modeling of the radiation sensitivity network: a biomarker discovery platform. Int. J. Radiat. Oncol. Biol. Phys. 75 (2), 497–505. 10.1016/j.ijrobp.2009.05.056 19735874 PMC2762403

[B17] Ettinger D. S. Wood D. E. Aisner D. L. Akerley W. Bauman J. R. Bharat A. (2021). NCCN guidelines insights: Non-small cell lung cancer, version 2.2021. J. Natl. Compr. Canc. Netw. 19 (3), 254–266. 10.6004/jnccn.2021.0013 33668021

[B18] Ferlay J. Colombet M. Soerjomataram I. Mathers C. Parkin D. M. PinerosM. (2019). Estimating the global cancer incidence and mortality in 2018: GLOBOCAN sources and methods. Int. J. Cancer 144 (8), 1941–1953. 10.1002/ijc.31937 30350310

[B19] Gao Z. Zhuang L. Chen Y. (2012). Effect and mechanism of gefitinib inhibition on non-small cell lung cancer radiosensitivity of HCC827 and H358 cell lines. Zhongguo Fei Ai Za Zhi 15 (6), 324–331. 10.3779/j.issn.1009-3419.2012.06.02 22681918 PMC6000299

[B20] Goldman M. J. Craft B. Hastie M. Repečka K. McDade F. Kamath A. (2020). Visualizing and interpreting cancer genomics data via the Xena platform. Nat. Biotechnol. 38 (6), 675–678. 10.1038/s41587-020-0546-8 32444850 PMC7386072

[B21] Goldstraw P. Chansky K. Crowley J. Rami-Porta R. Asamura H. Eberhardt W. E. E. (2016). The IASLC lung cancer staging project: proposals for revision of the TNM stage groupings in the forthcoming (eighth) edition of the TNM classification for lung cancer. J. Thorac. Oncol. 11 (1), 39–51. 10.1016/j.jtho.2015.09.009 26762738

[B22] Gu C. Shi X. Dai C. Shen F. Rocco G. Chen J. (2020). RNA m^6^A modification in cancers: molecular mechanisms and potential clinical applications. Innovation. 1 (3), 100066. 10.1016/j.xinn.2020.100066 34557726 PMC8454620

[B23] Hanash S. M. Bobek M. P. Rickman D. S. Williams T. Rouillard J. M. Kuick R. (2002). Integrating cancer genomics and proteomics in the post-genome era. Proteomics 2 (1), 69–75. 10.1002/1615-9861(200201)2:1<69::aid-prot69>3.0.co;2-8 11788993

[B24] Hanzelmann S. Castelo R. Guinney J. (2013). GSVA: gene set variation analysis for microarray and RNA-seq data. BMC Bioinforma. 14, 7. 10.1186/1471-2105-14-7 PMC3618321 23323831

[B25] He J. Zhang W. Li F. Yu Y. (2021). Development of metastasis-associated seven gene signature for predicting lung adenocarcinoma prognosis using single-cell RNA sequencing data. Math. Biosci. Eng. 18 (5), 5959–5977. 10.3934/mbe.2021298 34517518

[B26] Ho T. H. Kapur P. Joseph R. W. Serie D. J. Eckel-Passow J. E. Tong P. (2016). Loss of histone H3 lysine 36 trimethylation is associated with an increased risk of renal cell carcinoma-specific death. Mod. Pathol. 29 (1), 34–42. 10.1038/modpathol.2015.123 26516698 PMC4697879

[B27] Holgersson G. Bergström S. Bergqvist M. Nyman J. Hoye E. Helsing M. (2011). Swedish lung cancer radiation study group: predictive value of histology for radiotherapy response in patients with non-small cell lung cancer. Eur. J. Cancer 47 (16), 2415–2421. 10.1016/j.ejca.2011.06.011 21726999

[B28] Hörner-Rieber J. Bernhardt D. Dern J. König L. Adeberg S. Paul A. (2017). Histology of non-small cell lung cancer predicts the response to stereotactic body radiotherapy. Radiother. Oncol. 125 (2), 317–324. 10.1016/j.radonc.2017.08.029 28919006

[B29] Huang H. Weng H. Zhou K. Wu T. Zhao B. S. Sun M. (2019). Histone H3 trimethylation at lysine 36 guides m(6)A RNA modification co-transcriptionally. Nature 567 (7748), 414–419. 10.1038/s41586-019-1016-7 30867593 PMC6438714

[B30] Huang W. Chen T.-Q. Fang K. Zeng Z.-C. Ye H. Chen Y.-Q. (2021). N6-methyladenosine methyltransferases: functions, regulation, and clinical potential. J. Hematol. Oncol. 14 (1), 117. 10.1186/s13045-021-01129-8 34315512 PMC8313886

[B31] Karlsson M. Zhang C. Mear L. Zhong W. Digre A. Katona B. (2021). A single-cell type transcriptomics map of human tissues. Sci. Adv. 7 (31), eabh2169. 10.1126/sciadv.abh2169 34321199 PMC8318366

[B32] Katagiri Y. Jingu K. Yamamoto T. Matsushita H. Umezawa R. Ishikawa Y. (2021). Differences in patterns of recurrence of squamous cell carcinoma and adenocarcinoma after radiotherapy for stage III non-small cell lung cancer. Jpn. J. Radiol. 39 (6), 611–617. 10.1007/s11604-021-01091-y 33484424

[B33] Kellerer A. M. Rossi H. H. (1978). A generalized formulation of dual radiation action. Radiat. Res. 75 (3), 471. 10.2307/3574835 22870971

[B34] Kim N. Kim H. K. Lee K. Hong Y. Cho J. H. Choi J. W. (2020). Single-cell RNA sequencing demonstrates the molecular and cellular reprogramming of metastatic lung adenocarcinoma. Nat. Commun. 11 (1), 2285. 10.1038/s41467-020-16164-1 32385277 PMC7210975

[B35] Kocakavuk E. Anderson K. J. Varn F. S. Johnson K. C. Amin S. B. Sulman E. P. (2021). Radiotherapy is associated with a deletion signature that contributes to poor outcomes in patients with cancer. Nat. Genet. 53 (7), 1088–1096. 10.1038/s41588-021-00874-3 34045764 PMC8483261

[B36] Kumari S. Muthusamy S. (2020). SETD2 as a regulator of N6-methyladenosine RNA methylation and modifiers in cancer. Eur. J. Cancer Prev. 29 (6), 556–564. 10.1097/cej.0000000000000587 33021769

[B37] Kundaje A. Meuleman W. Ernst J. Bilenky M. Yen A. Heravi-Moussavi A. (2015). Integrative analysis of 111 reference human epigenomes. Nature 518 (7539), 317–330. 10.1038/nature14248 25693563 PMC4530010

[B38] Lambrechts D. Wauters E. Boeckx B. Aibar S. Nittner D. Burton O. (2018). Phenotype molding of stromal cells in the lung tumor microenvironment. Nat. Med. 24 (8), 1277–1289. 10.1038/s41591-018-0096-5 29988129

[B39] Langfelder P. Horvath S. (2008). WGCNA: an R package for weighted correlation network analysis. BMC Bioinform. 9, 559. 10.1186/1471-2105-9-559 PMC2631488 19114008

[B40] Lê S. Josse J. Husson F. (2008). FactoMineR: an R package for multivariate analysis. J. Stat. Softw. 25, 1. 10.18637/jss.v025.i01

[B41] Leiserson M. D. Vandin F. Wu H. T. Dobson J. R. Eldridge J. V. Thomas J. L. (2015). Pan-cancer network analysis identifies combinations of rare somatic mutations across pathways and protein complexes. Nat. Genet. 47 (2), 106–114. 10.1038/ng.3168 25501392 PMC4444046

[B42] Li T. Fan J. Wang B. Traugh N. Chen Q. Liu J. S. (2017). TIMER: a web server for comprehensive analysis of tumor-infiltrating immune cells. Cancer Res. 77 (21), e108–e110. 10.1158/0008-5472.can-17-0307 29092952 PMC6042652

[B43] Li J. Rao B. Yang J. Liu L. Huang M. Liu X. (2020). Dysregulated m6A-related regulators are associated with tumor metastasis and poor prognosis in osteosarcoma. Front. Oncol. 10, 769. 10.3389/fonc.2020.00769 32582536 PMC7280491

[B44] Liu T. Ortiz J. A. Taing L. Meyer C. A. Lee B. Zhang Y. (2011). Cistrome: an integrative platform for transcriptional regulation studies. Genome Biol. 12 (8), R83. 10.1186/gb-2011-12-8-r83 21859476 PMC3245621

[B45] Liu W. Fu Q. An H. Chang Y. Zhang W. Zhu Y. (2015). Decreased expression of SETD2 predicts unfavorable prognosis in patients with nonmetastatic clear-cell renal cell carcinoma. Med. (Baltimore) 94 (45), e2004. 10.1097/md.0000000000002004 PMC4912287 26559293

[B46] Liu Y. Burness M. L. Martin-Trevino R. Guy J. Bai S. Harouaka R. (2017). RAD51 mediates resistance of cancer stem cells to PARP inhibition in triple-negative breast cancer. Clin. Cancer Res. 23 (2), 514–522. 10.1158/1078-0432.CCR-15-1348 28034904

[B47] Mar B. G. Chu S. H. Kahn J. D. Krivtsov A. V. Koche R. Castellano C. A. (2017). SETD2 alterations impair DNA damage recognition and lead to resistance to chemotherapy in leukemia. Blood 130 (24), 2631–2641. 10.1182/blood-2017-03-775569 29018079 PMC5731084

[B48] McAleese J. Taylor A. Walls G. M. Hanna G. G. (2019). Differential relapse patterns for non-small cell lung cancer subtypes adenocarcinoma and squamous cell carcinoma: implications for radiation oncology. Clin. Oncol. 31 (10), 711–719. 10.1016/j.clon.2019.07.008 31351746

[B49] McInnes L. Healy J. Saul N. Grossberger L. (2018). UMAP: uniform manifold approximation and projection. J. Open Source Softw. 3, 861. 10.21105/joss.00861

[B50] Miyakawa A. Shibamoto Y. Kosaki K. Hashizume C. (2013). Early response and local control of stage I non-small-cell lung cancer after stereotactic radiotherapy: difference by histology. Cancer Sci. 104 (1), 130–134. 10.1111/cas.12048 23095036 PMC7657123

[B51] Nakayama Y. Hayakawa K. Mitsuhashi N. Saito Y. Niibe H. (1997). Long-term survivors of non-small cell lung cancer after radiation therapy: the significance of histological type. Anticancer Res. 17 (4), 2769–2773. 9252713

[B52] Oleinick N. L. Biswas T. Patel R. Tao M. Patel R. Weeks L. (2016). Radiosensitization of non-small-cell lung cancer cells and xenografts by the interactive effects of pemetrexed and methoxyamine. Radiother. Oncol. 121 (2), 335–341. 10.1016/j.radonc.2016.10.007 27838149

[B53] Pahuja S. Beumer J. H. Appleman L. J. Tawbi H. A.-H. Stoller R. G. Lee J. J. (2014). Outcome of BRCA 1/2-mutated (BRCA+) and triple-negative, BRCA wild type (BRCA-wt) breast cancer patients in a phase I study of single-agent veliparib (V). J. Clin. Oncol. 32 (26), 135. 10.1200/jco.2014.32.26_suppl.135

[B54] Park J. G. Paul S. Briones N. Zeng J. Gillis K. Wallstrom G. (2017). Developing human radiation biodosimetry models: testing cross-species conversion approaches using an *Ex Vivo* model system. Radiat. Res. 187 (6), 708–721. 10.1667/rr14655.1 28328310 PMC5996993

[B55] Peng Q. Weng K. Li S. Xu R. Wang Y. Wu Y. (2021). A perspective of epigenetic regulation in radiotherapy. Front. Cell Dev. Biol. 9, 624312. 10.3389/fcell.2021.624312 33681204 PMC7930394

[B56] Pfister S. X. Ahrabi S. Zalmas L.-P. Sarkar S. Aymard F. Bachrati C. Z. (2014). SETD2-dependent histone H3K36 trimethylation is required for homologous recombination repair and genome stability. Cell Rep. 7 (6), 2006–2018. 10.1016/j.celrep.2014.05.026 24931610 PMC4074340

[B57] Pollom E. L. Qian Y. Durkee B. Y. von Eyben R. Maxim P. G. Shultz D. B. (2016). Hypofractionated intensity-modulated radiotherapy for patients with non-small-cell lung cancer. Clin. Lung Cancer 17 (6), 588–594. 10.1016/j.cllc.2016.05.024 27378172

[B58] Pujade-Lauraine E. Ledermann J. A. Selle F. Gebski V. Penson R. T. Oza A. M. (2017). Olaparib tablets as maintenance therapy in patients with platinum-sensitive, relapsed ovarian cancer and a BRCA1/2 mutation (SOLO2/ENGOT-Ov21): a double-blind, randomised, placebo-controlled, phase 3 trial. Lancet. Oncol. 18 (9), 1274–1284. 10.1016/s1470-2045(17)30469-2 28754483

[B59] Reinhold W. C. Reimers M. A. Lorenzi P. Ho J. Shankavaram U. T. Ziegler M. S. (2010). Multifactorial regulation of E-cadherin expression: an integrative study. Mol. Cancer Ther. 9 (1), 1–16. 10.1158/1535-7163.mct-09-0321 20053763 PMC2821037

[B60] Satija R. Farrell J. A. Gennert D. Schier A. F. Regev A. (2015). Spatial reconstruction of single-cell gene expression data. Nat. Biotechnol. 33 (5), 495–502. 10.1038/nbt.3192 25867923 PMC4430369

[B61] Scott J. G. Berglund A. Schell M. J. Mihaylov I. Fulp W. J. Yue B. (2017). A genome-based model for adjusting radiotherapy dose (GARD): a retrospective, cohort-based study. Lancet. Oncol. 18 (2), 202–211. 10.1016/S1470-2045(16)30648-9 27993569 PMC7771305

[B62] Shannon P. Markiel A. Ozier O. Baliga N. S. Wang J. T. Ramage D. (2003). Cytoscape: a software environment for integrated models of biomolecular interaction networks. Genome Res. 13 (11), 2498–2504. 10.1101/gr.1239303 14597658 PMC403769

[B63] Shirbhate E. Patel P. Patel V. K. Veerasamy R. Sharma P. C. Rajak H. (2020). The combination of histone deacetylase inhibitors and radiotherapy: a promising novel approach for cancer treatment. Future Oncol. 16 (30), 2457–2469. 10.2217/fon-2020-0385 32815411

[B64] Skucha A. Ebner J. Grebien F. (2019). Roles of SETD2 in leukemia-transcription, DNA-damage, and beyond. Int. J. Mol. Sci. 20 (5), E1029. 10.3390/ijms20051029 30818762 PMC6429614

[B65] Slade D. (2020). PARP and PARG inhibitors in cancer treatment. Genes Dev. 34 (5-6), 360–394. 10.1101/gad.334516.119 32029455 PMC7050487

[B66] Sung H. Ferlay J. Siegel R. L. Laversanne M. Soerjomataram I. Jemal A. (2021). Global cancer statistics 2020: GLOBOCAN estimates of incidence and mortality worldwide for 36 cancers in 185 countries. CA Cancer J. Clin. 71 (3), 209–249. 10.3322/caac.21660 33538338

[B67] Szklarczyk D. Morris J. H. Cook H. Kuhn M. Wyder S. Simonovic M. (2017). The STRING database in 2017: quality-controlled protein-protein association networks, made broadly accessible. Nucleic Acids Res. 45 (D1), D362–D368. 10.1093/nar/gkw937 27924014 PMC5210637

[B68] Taketo K. Konno M. Asai A. Koseki J. Toratani M. Satoh T. (2018). The epitranscriptome m^6^A writer METTL3 promotes chemo- and radioresistance in pancreatic cancer cells. Int. J. Oncol. 52 (2), 621–629. 10.3892/ijo.2017.4219 29345285

[B69] Tang Z. Li C. Kang B. Gao G. Li C. Zhang Z. (2017a). GEPIA: a web server for cancer and normal gene expression profiling and interactive analyses. Nucleic Acids Res. 45 (W1), W98–W102. 10.1093/nar/gkx247 28407145 PMC5570223

[B70] Tang Z. Zeng Q. Li Y. Zhang X. Suto M. J. Xu B. (2017b). Predicting radiotherapy response for patients with soft tissue sarcoma by developing a molecular signature. Oncol. Rep. 38 (5), 2814–2824. 10.3892/or.2017.5999 29048650 PMC5780036

[B71] Torres-Roca J. F. Eschrich S. Zhao H. Bloom G. Sung J. McCarthy S. (2005). Prediction of radiation sensitivity using a gene expression classifier. Cancer Res. 65 (16), 7169–7176. 10.1158/0008-5472.can-05-0656 16103067

[B72] Visvanathan A. Patil V. Arora A. Hegde A. S. Arivazhagan A. Santosh V. (2018). Essential role of METTL3-mediated m(6)A modification in glioma stem-like cells maintenance and radioresistance. Oncogene 37 (4), 522–533. 10.1038/onc.2017.351 28991227

[B73] Wang S. Sun H. Ma J. Zang C. Wang C. Wang J. (2013). Target analysis by integration of transcriptome and ChIP-seq data with BETA. Nat. Protoc. 8 (12), 2502–2515. 10.1038/nprot.2013.150 24263090 PMC4135175

[B74] Wu C. Guo E. Ming J. Sun W. Nie X. Sun L. (2020). Radiation-induced DNMT3B promotes radioresistance in nasopharyngeal carcinoma through methylation of p53 and p21. Mol. Ther. Oncolytics 17, 306–319. 10.1016/j.omto.2020.04.007 32382655 PMC7200625

[B75] Wu P. Fang X. Liu Y. Tang Y. Wang W. Li X. (2021). N6-methyladenosine modification of circCUX1 confers radioresistance of hypopharyngeal squamous cell carcinoma through caspase1 pathway. Cell Death Dis. 12 (4), 298. 10.1038/s41419-021-03558-2 33741902 PMC7979824

[B76] Xu Y. Su G. H. Ma D. Xiao Y. Shao Z. M. Jiang Y. Z. (2021). Technological advances in cancer immunity: from immunogenomics to single-cell analysis and artificial intelligence. Signal Transduct. Target. Ther. 6 (1), 312. 10.1038/s41392-021-00729-7 34417437 PMC8377461

[B77] Xue G. Ren Z. Chen Y. Zhu J. Du Y. Pan D. (2015). A feedback regulation between miR-145 and DNA methyltransferase 3b in prostate cancer cell and their responses to irradiation. Cancer Lett. 361 (1), 121–127. 10.1016/j.canlet.2015.02.046 25749421

[B78] Yu G. Wang L. G. Han Y. He Q. Y. (2012). clusterProfiler: an R package for comparing biological themes among gene clusters. OMICS 16 (5), 284–287. 10.1089/omi.2011.0118 22455463 PMC3339379

[B79] Yu G. Wang L. G. He Q. Y. (2015). ChIPseeker: an R/bioconductor package for ChIP peak annotation, comparison and visualization. Bioinformatics 31 (14), 2382–2383. 10.1093/bioinformatics/btv145 25765347

[B80] Zeng Z. Gao Y. Li J. Zhang J. Li Y. He F. (Forthcoming 2022). SETD2 mediates immunotherapy and radiotherapy efficacy via regulating DNA damage responses and genomic stability in lung adenocarcinoma. Genes Dis. 10.1016/j.gendis.2022.02.016 PMC10201589 37223520

[B81] Zhong X. Luo G. Zhou X. Luo W. Wu X. Zhong R. (2016). Rad51 in regulating the radiosensitivity of non-small cell lung cancer with different epidermal growth factor receptor mutation status. Thorac. Cancer 7 (1), 50–60. 10.1111/1759-7714.12274 26816539 PMC4718133

