# Supplementary material for: SETD2 regulates gene transcription patterns and is associated with radiosensitivity in lung adenocarcinoma
Source: Front Genet. 2022 Aug 10;13:935601. doi: 10.3389/fgene.2022.935601 (PMC9399372; doi:10.3389/fgene.2022.935601)
Supplement: Supplementary file 2 [file Table1.DOCX]

**Supplementary Table 1. The basic information of included datasets.**

| **Datasets** | **Number** | **Data Type** | **Histological Type** | **Reference** |
| --- | --- | --- | --- | --- |
| GSE20549 | 42 | Microarray | H460 and H1299 cells | [https://www.ncbi.nlm.nih.gov/geo/query/acc.cgi?acc=GSE20549](https://www.ncbi.nlm.nih.gov/geo/query/acc.cgi?acc=GSE20549) |
| GSE32036 | 10 | Microarray | NSCLC cells | PMID: 23091115 |
| GSE57083 | 16 | Microarray | NSCLC cells | [https://www.ncbi.nlm.nih.gov/geo/query/acc.cgi?acc=GSE57083](https://www.ncbi.nlm.nih.gov/geo/query/acc.cgi?acc=GSE57083) |
| GSE5949 | 59 | Microarray | Pan-cancer cells | PMID: 20053763 |
| GSE50081 | 127 | Microarray | LUAD cells | PMID: 24305008 |
| GSE3141 | 58 | Microarray | LUAD cells | PMID: 16273092 |
| GSE121949 | 16 | RNA-seq | HepG2 cells | PMID: 30867593 |
| The Cancer Genome Atlas | 10953 | RNA-seq; Methylation 450K BeadChip;ATAC-seq | Pan-cancer cells | PMID: 26704973; PMID: 32444850; PMID: 30561551 |
| GSE110318 | 8 | ChIP-seq | HepG2 cells | PMID: 30867593 |
| Roadmap | 6 | ChIP-seq | Lung cancer cells | PMID: 25693563 |
| GSE131907 | 45149 | scRNA-seq | LUAD cells | PMID: 32385277 |
