# Supplementary material for: SETD2 regulates gene transcription patterns and is associated with radiosensitivity in lung adenocarcinoma
Source: Front Genet. 2022 Aug 10;13:935601. doi: 10.3389/fgene.2022.935601 (PMC9399372; doi:10.3389/fgene.2022.935601)
Supplement: Supplementary file 12 [file Table2.DOCX]

**Supplementary Table 2. SF2 values from previous laboratory studies.**

| **Cell lines** | **SF2 value** | **Tumor type** |
| --- | --- | --- |
| A549 | 0.61 | NSCLC |
| EKVX | 0.7 | NSCLC |
| H23 | 0.086 | NSCLC |
| H358 | 0.433 | NSCLC |
| H460 | 0.84 | NSCLC |
| H820 | 0.4 | NSCLC |
| H1299 | 0.601 | NSCLC |
| HCC827 | 0.242 | NSCLC |
| HOP-62 | 0.164 | NSCLC |
| HOP-92 | 0.43 | NSCLC |
| H226 | 0.631 | NSCLC |
| H322 | 0.65 | NSCLC |
| H522 | 0.43 | NSCLC |
| HS578T | 0.79 | other pan-cancer |
| MDAMB231 | 0.82 | other pan-cancer |
| HCT116 | 0.38 | other pan-cancer |
| HCT15 | 0.4 | other pan-cancer |
| SW620 | 0.62 | other pan-cancer |
| CCRFCEM | 0.185 | other pan-cancer |
| HL60 | 0.315 | other pan-cancer |
| MOLT4 | 0.05 | other pan-cancer |
| SKMEL2 | 0.66 | other pan-cancer |
| OVCAR5 | 0.408 | other pan-cancer |
| SN12C | 0.62 | other pan-cancer |
| BT549 | 0.632 | other pan-cancer |
| MCF7 | 0.576 | other pan-cancer |
| MDAMB435 | 0.1795 | other pan-cancer |
| T47D | 0.52 | other pan-cancer |
| SF268 | 0.45 | other pan-cancer |
| SF539 | 0.82 | other pan-cancer |
| SNB19 | 0.43 | other pan-cancer |
| SNB75 | 0.55 | other pan-cancer |
| U251 | 0.57 | other pan-cancer |
| COLO205 | 0.69 | other pan-cancer |
| HCC-2998 | 0.44 | other pan-cancer |
| HT29 | 0.79 | other pan-cancer |
| KM12 | 0.42 | other pan-cancer |
| LOXIMVI | 0.68 | other pan-cancer |
| M14 | 0.42 | other pan-cancer |
| MALME3M | 0.8 | other pan-cancer |
| SKMEL28 | 0.74 | other pan-cancer |
| SKMEL5 | 0.72 | other pan-cancer |
| UACC257 | 0.48 | other pan-cancer |
| UACC62 | 0.52 | other pan-cancer |
| OVCAR3 | 0.55 | other pan-cancer |
| OVCAR4 | 0.29 | other pan-cancer |
| OVCAR8 | 0.6 | other pan-cancer |
| SKOV3 | 0.9 | other pan-cancer |
| DU145 | 0.52 | other pan-cancer |
| PC3 | 0.484 | other pan-cancer |
| 7860 | 0.66 | other pan-cancer |
| A498 | 0.61 | other pan-cancer |
| ACHN | 0.72 | other pan-cancer |
| CAKI1 | 0.37 | other pan-cancer |
| UO31 | 0.62 | other pan-cancer |
| MCF7ADRr | 0.561 | other pan-cancer |
| MDN | 0.7 | other pan-cancer |
| SF295 | 0.73 | other pan-cancer |
| K562 | 0.0505 | other pan-cancer |
| RPMI8266 | 0.099 | other pan-cancer |
| SR | 0.068 | other pan-cancer |
| IGROV1 | 0.389 | other pan-cancer |
| RXF393 | 0.67 | other pan-cancer |
| TK10 | 0.518 | other pan-cancer |
