# Supplementary material for: SETD2 regulates gene transcription patterns and is associated with radiosensitivity in lung adenocarcinoma
Source: Front Genet. 2022 Aug 10;13:935601. doi: 10.3389/fgene.2022.935601 (PMC9399372; doi:10.3389/fgene.2022.935601)
Supplement: Supplementary file 13 [file DataSheet1.PDF]

Supplementary Material

The general flow of the methods

In this study, we developed a novel key gene identification workflow. Specifically, we collected 42 NSCLC cell samples from GSE20549 (Clough and Barrett, 2016) with 6 time points (0, 2, 4, 8, 12, and 24 h) after 2 Gy IR. Next was the initial screening: ANOVA was performed for each gene across the 6 time points, and the significant genes ($p < 0.05$) were selected for further analysis. We then performed STEM (Ernst and Bar-Joseph, 2006) analysis to identify 11 time-course gene clusters (FDR $q < 0.01$). We used PCA to extract the eigenvalues of each cluster. Next, we performed Spearman's correlation between the eigenvalue of gene clusters and SF2. The 5 clusters with large correlation coefficients were considered to be SF2-related gene clusters. Enrichment analysis was used to indicate the biological pathways related to each cluster. We then further screened the genes of these 5 clusters. First, we used RF (Breiman, 2001) and SGLQ model algorithms to fit SF2 in each gene expression. Genes with lncMSE less than 0 in the RF model and P greater than 0.05 in the SGLQ model (poor performance) were excluded. Next, we constructed the 4-level network, containing the mutation, CNA, RNA, and protein subnetworks from TCGA pan-cancer data using the remaining genes. In the network, genes with the degree, betweenness, and closeness values all greater than the median were considered to be key genes. Enrichment analysis was used to indicate the biological pathways related to these genes. The histone H3K36 methylation was significant in enrichment analysis. Therefore, we next performed Spearman's correlation analysis between H3K36 methylation regulatory genes (SETD2, SETD3, NSD1, PAXIP1, BRD4, IWS1, SETMAR, SMYD2, ASH1L) and SF2. SETD2 was the only gene with $p < 0.05$. We expected this workflow to contribute to other related studies.

Reference

Breiman, L. (2001). Random Forests. *Machine Learning* 45(1)**,** 5-32. doi: 10.1023/A:1010933404324.

Clough, E., and Barrett, T. (2016). The Gene Expression Omnibus Database. *Methods Mol Biol* 1418**,** 93-110. doi: 10.1007/978-1-4939-3578-9_5.

Ernst, J., and Bar-Joseph, Z. (2006). STEM: a tool for the analysis of short time series gene expression data. *BMC Bioinformatics* 7(1)**,** 191. doi: 10.1186/1471-2105-7-191.

Shannon, P., Markiel, A., Ozier, O., Baliga, N.S., Wang, J.T., Ramage, D., et al. (2003). Cytoscape: a software environment for integrated models of biomolecular interaction networks. *Genome Res* 13(11)**,** 2498-2504. doi: 10.1101/gr.1239303.
