# Supplementary material for: SETD2 regulates gene transcription patterns and is associated with radiosensitivity in lung adenocarcinoma
Source: Front Genet. 2022 Aug 10;13:935601. doi: 10.3389/fgene.2022.935601 (PMC9399372; doi:10.3389/fgene.2022.935601)
Supplement: Supplementary file 14 [file Table3.DOCX]

**Supplementary Table 3. Multivariate Cox regression with RBM15 * SETD2 interaction.**

|  | **HR** | **P** |
| --- | --- | --- |
| SETD2 | 0.517677 | 0.00629 |
| Age | 1.013206 | 8.82E-02 |
| Stage | 1.654581 | 6.32E-12 |
| Gender | 1.001567 | 9.92E-01 |
| Interaction term | 1.145387 | 0.02046 |
