# Supplementary material for: SETD2 regulates gene transcription patterns and is associated with radiosensitivity in lung adenocarcinoma
Source: Front Genet. 2022 Aug 10;13:935601. doi: 10.3389/fgene.2022.935601 (PMC9399372; doi:10.3389/fgene.2022.935601)
Supplement: Supplementary file 15 [file Table4.DOCX]

**Supplementary Table 4. Multivariate Cox regression with YTHDF3 * SETD2 interaction.**

|  | **HR** | **P** |
| --- | --- | --- |
| SETD2 | 0.6035 | 0.065 |
| Age | 1.01198 | 1.20E-01 |
| Stage | 1.64632 | 1.42E-11 |
| Gender | 0.96845 | 8.35E-01 |
| Interaction term | 1.05998 | 0.184 |
